# Effect of Thickener Rheology on Bolus Cohesivity in Dysphagia Management

**DOI:** 10.1111/jtxs.70107

**Published:** 2026-08-23

**Authors:** Mats Stading, Johanna Eckardt

**Affiliations:** ^1^ RISE Research Institutes of Sweden Gothenburg Sweden; ^2^ Chalmers University of Technology Gothenburg Sweden

**Keywords:** bolus; cohesivity, dysphagia, Gothenburg throat model, swallowing

## Abstract

Dysphagia caused by dysfunction is commonly managed by thickened beverages, yet fluids with similar viscosity may differ markedly in terms of swallowing safety. This study investigates how thickening mechanisms such as viscosity level, elasticity, and shear‐thinning affect bolus cohesivity and aspiration risk. Model fluids with identical shear viscosity at 50 s^−1^ of 0.15 Pa s and IDDSI level 2 were formulated: Newtonian (maltodextrin), Boger (maltodextrin + xanthan, elastic), and shear‐thinning (xanthan). Their flow behavior was evaluated in a swallowing simulator (“The Gothenburg Throat”) replicating healthy and impaired conditions, combined with shear and extensional rheometry. Despite identical shear viscosity, marked differences in flow were observed. Newtonian fluids showed insufficient cohesivity and allowed aspiration under impaired conditions, whereas both elastic (Boger) and shear‐thinning fluids maintained cohesive boluses and prevented airway entry. Elastic and extensional properties enhance bolus integrity, reducing droplet breakup. Analysis using dimensionless numbers indicated that neither viscosity nor inertia alone predicts performance; instead, a combined “bolus cohesivity number” incorporating viscous, elastic, inertial, and surface forces correlated with aspiration outcomes. These results demonstrate that the mechanism of thickening is critical: elasticity and shear‐thinning improve swallowing safety beyond viscosity alone, which has strong implications for the design and selection of dysphagia thickeners.

## Graphical abstract

Thickened liquids have different rheology: Newtonian ones lack cohesivity and cause aspiration, while elastic and shear‐thinning types form cohesive boluses preventing airway entry. Elasticity and extensional effects govern bolus integrity, so shear viscosity alone is insufficient; a “bolus cohesivity number” integrating viscous, elastic, inertial, and surface forces predicts aspiration.

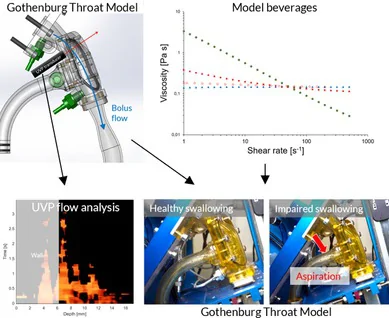

## 1 Introduction

Swallowing disorders, or dysphagia, affect 10%–30% of people aged 65 and above (Barczi et al. 2000) due to factors such as degenerative diseases, side effects of medication or simply age‐related impairment of physiological oropharyngeal function. Age‐related dysphagia is rarely curable and managed by texture‐modified foods and thickened beverages (Ekberg 2019). The increased viscosity of the beverage induces slower flow, which allows sufficient time also for an age‐impaired physiology to swallow safely (Newman et al. 2016). A specific factor to avoid fluid entering the airway, that is, aspiration, is to assure cohesivity of the bolus so it does not break up into droplets.

Dysphagia caused by dysfunction is clinically diagnosed mainly by two analytical methods: (i) X‐ray video‐fluoroscopy allows real‐time assessment of the anatomy and physiology of the swallowing apparatus during bolus transport (Ekberg 2019). (ii) Fiber Optic Endoscopic Evaluation of Swallowing (FEES) is used to optically examine abnormal swallowing function. FEES is a useful bedside swallowing examination to visualize the anatomy of the pharynx and larynx without X‐ray exposure and is highly sensitive for detection of aspiration after swallowing (Benjapornlert et al. 2020). Both methods are used clinically as the gold standards for diagnosing dysphagia. In addition, other methods such as questionnaires and observation swallow of different fluids are used clinically.

When a person is diagnosed with dysphagia, the management of drinking is mainly through thickened beverages where the level of thickening is expressed through the IDDSI‐level (International Dysphagia Diet Standardization Initiative) (Cichero et al. 2017). For beverages, the scale goes from 0 (not thickened) to 4 (extremely thickened), where IDDSI 1 and 2 are common. The levels 1–3 are clinically tested by letting the beverage drip out of a syringe with specified dimension for 10 s (Steele et al. 2024). The rheology of this test has been thoroughly evaluated and translated to Power law parameters n and K obtained by viscometry (Lecanu et al. 2024). The study shows that several fluids with a wide range of shear‐thinning can still fall into a specific thickness level. However, when considering other fluid properties such as elasticity, the classification becomes much more complicated (Lecanu et al. 2026). It should also be noted that overly viscous or elastic fluids can impair swallowing due to fragmentation with remaining pieces in the pharynx or excessive muscle force required to initiate swallowing.

Cohesiveness has been discussed by several authors and is easy to understand intuitively but has not, to our knowledge, been defined in physical terms (Burbidge et al. 2016; Chen and Lolivret 2011; Gallegos et al. 2023; Hadde et al. 2019; Nyström et al. 2015; McFarland et al. 2026; Wang et al. 2026). By modeling, it has been shown for Newtonian fluids that fragmentation (inverse cohesiveness) increased with bolus volume and velocity and decreased with viscosity (McFarland et al. 2026). Burbridge and co‐workers have also analyzed fluid mechanics of swallowing for Newtonian fluids (Burbidge et al. 2016). They concluded that viscosity is important for both the oral phase to avoid spill into the pharynx (manipulation of the bolus before swallowing) and for slowing down the flow during the pharyngeal phase. They also highlight the inertial effects (i.e., density) especially for fast flow. Water commonly exhibits turbulent flow, whereas thickened fluids flow in a laminar manner. Several authors indicate that fluid elasticity is important for cohesivity even though it remains to be proven in terms of fluid dynamics (Chen and Lolivret 2011; Gallegos et al. 2023; Hadde et al. 2019; Nyström et al. 2015; Wang et al. 2026). In anticipation of a stricter definition, cohesiveness will here be used in a loose sense meaning how well a bolus keeps together in time and space.

Fluid rheology can be measured non‐invasively orally over short distances (Sepehri et al. 2023) but this method is still in the experimental stage. As the complete oropharyngeal flow is of interest, model fluids have been used to capture the influence of different rheological parameters on swallowing. Newtonian fluids are commonly compared to Boger fluids to identify effects of fluid elasticity (Nyström et al. 2015). In a Boger fluid, a small amount of polymer is added to a high‐viscous Newtonian fluid which shields the shear‐thinning, thus keeping an almost constant shear viscosity in addition to being elastic (Boger 1977). The effect of elasticity is often contradicted by shear‐thinning, and such fluids are also interesting to include as model fluids. The shear viscosity can then only be compared for one shear rate, and for swallowing, 50 s^−1^ is often chosen although real shear rates in the swallowing process have been reported in the range 10–900 s^−1^ (Gallegos et al. 2023; Qazi et al. 2019).

Edible Boger fluids were first developed for sensory analysis (Koliandris et al. 2011). Edible model fluids can be formulated from viscous maltodextrin solutions with added xanthan for elasticity (Nyström et al. 2015; Qazi et al. 2020). For high viscosity, the effect of shear‐thinning of xanthan can be masked by the Newtonian fluid, whereas for lower viscosity, the addition is a balance between adding sufficient xanthan to induce elasticity and at the same time keeping the addition low enough to suppress shear‐thinning (Nyström et al. 2015). For a Power Law fluid, this means keeping the shear thinning index n as close to *n* = 1 as possible. The use of polyacrylamide instead of xanthan produces Boger fluids with more constant shear viscosity, but polyacrylamide is not edible (James 2009).

The aim of the present study was to determine which effect of thickening is most important for bolus cohesivity and to avoid aspiration. Model fluids were used to specifically study the effects of Newtonian, elastic, and shear‐thinning mechanisms.

## 2 Materials and Methods

### 2.1 Materials

The model fluids used in this study were prepared using maltodextrin Glucidex IT 19 (Roquette, Lestrem, France) and xanthan Grindstedt Clear 80 (IFF, Brabrand, Denmark). The ingredients were food grade to allow use of the same model fluids in sensory studies. The fluids were for the same reason mixed in a sugar‐free lemonade (Fun Light Lemonade, Orkla Foods, Oslo Norway). Polyacrylamide (PAA) 5–6 × 10^6^ Da (Thermo Fischer Scientific, 178042500, Waltham, USA) was used as a non‐edible reference instead of xanthan.

### 2.2 Model Fluids

Fun Light was mixed with water 1 + 9 according to specifications. Stock solutions of 60% w/w maltodextrin and 1% w/w xanthan in diluted Fun Light were mixed and stirred overnight on a magnetic stirrer. The stock solutions were then mixed into model fluids (Koliandris et al. 2011; Nyström et al. 2015) to fulfill two criteria (i) to have IDDSI level 2 (Cichero et al. 2017); and (ii) to have shear viscosity of 0.15 Pa s at 50 s^−1^. The mixed fluids were stirred overnight. The three fluids were:

Newtonian: 53.7% w/w maltodextrin.

Boger: 50% w/w maltodextrin and 0.045% w/w xanthan.

Shear‐thinning: 0.45% xanthan. The edible Boger fluid was compared to one with PAA instead of xanthan mixed as follows:

Boger_PAA_: 51.5% w/w maltodextrin and 0.06% w/w PAA.

### 2.3 Swallowing Model

**FIGURE 1:**
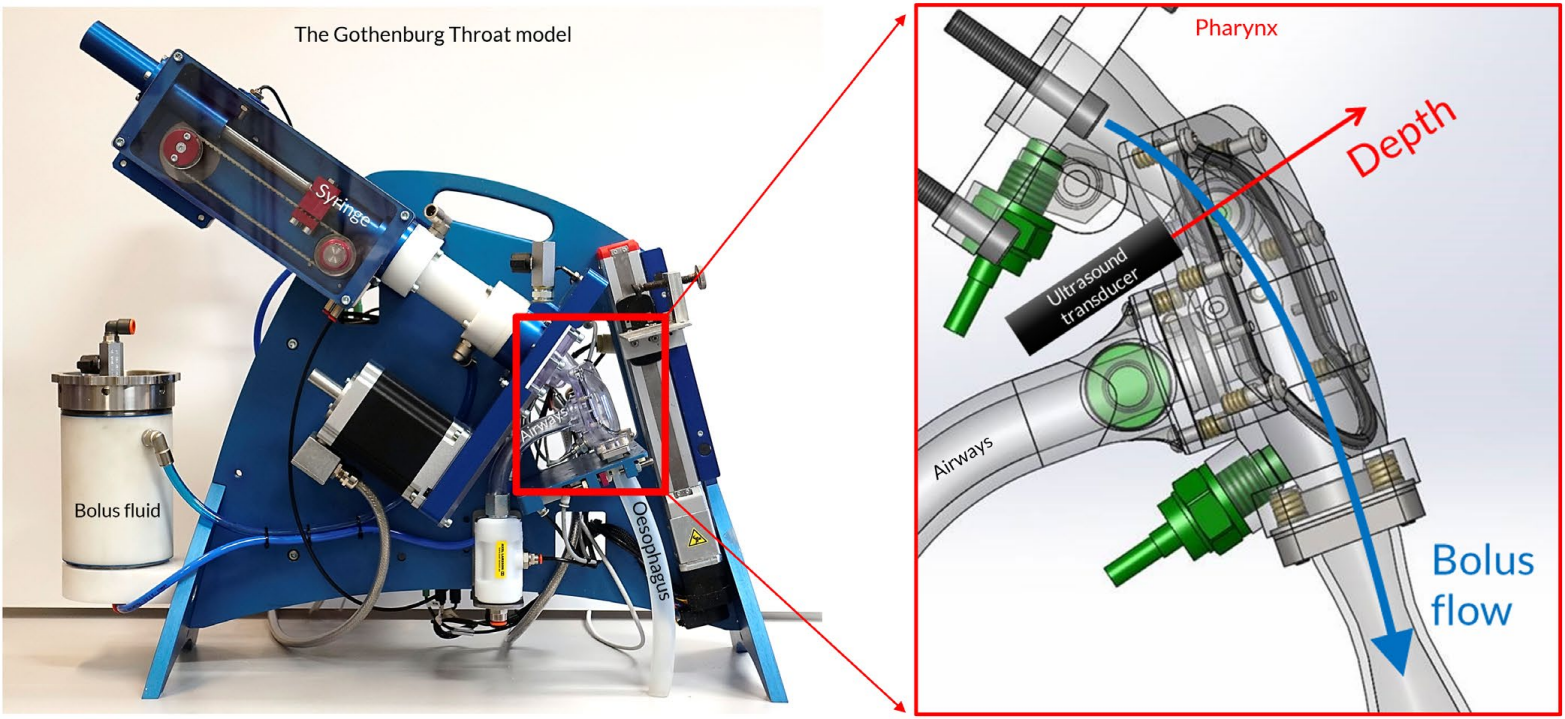
Overview of the swallowing simulator, the Gothenburg Throat model. The expanded schematic shows the pharynx section of the simulator with the ultrasonic transducer in black and pressure sensors in green.

The swallowing simulator, referred to as the Gothenburg Throat, has been described in detail elsewhere (Stading et al. 2019). A brief overview is provided here to introduce it to the reader (see Figure 1).

The test fluid is stored in a tank connected to the simulator and is delivered to a syringe pump representing the oral phase of swallowing. A bolus of predefined volume and injection velocity is introduced into the model pharynx, thereby mimicking the propulsive action of the tongue. During filling of the syringe, a slide valve remains closed to prevent gravity‐driven flow. The valve is opened only momentarily during bolus delivery, ensuring that flow is generated exclusively by the thrust applied by the syringe.

Anatomical structures are represented by mechanical components: two valves simulate the openings to the trachea and nasopharynx, a movable epiglottis closes during swallowing, and a clamping valve represents the upper esophageal sphincter (UES). The UES remains closed at the start of each swallow and opens transiently to allow bolus passage, coinciding with the closure of the epiglottis from its initial open position. The epiglottis does not form a hermetic seal over the laryngeal inlet.

As the bolus traverses the pharyngeal model, velocity profiles are measured using ultrasound velocity profiling, UVP, (Incipientus Ultrasound Flow Technologies, Västra Frölunda, Sweden) while pressure transducers record pressures at four predefined locations.

### 2.4 Device Settings

In the present study, the bolus volume was set to 30 ± 2 mL, which corresponds to a substantial gulp as we do when we drink. The ultrasonic transducer was placed above the epiglottis as shown in Figure 1. Two digital cameras (DSC‐RX100, Sony, Tokyo, Japan), positioned in front of and on the side of the pharynx part of the swallowing simulator to record the bolus motion. The piston speed, that is, the initial bolus speed was set to 0.30 ± 0.03 m/s and was kept constant independent of fluid rheology. For healthy swallowing the epiglottis closed immediately on initiation of swallowing, the airways (trachea) were closed and the UES open. For impaired swallowing, the closing of the epiglottis was delayed 1 s and the airways and UES were open. The ultrasonic transducer gives velocities along a beamline through the center of the pharynx as a function of depth and time as shown in Figure 1 (Qazi et al. 2020; Wiklund et al. 2007).

During bolus ejection, image acquisition was performed at 100 frames s^−1^. The recorded videos were analyzed and edited using Premiere (Adobe, San Jose, USA).

### 2.5 Shear Rheometry

Viscometry was performed using an ARES‐G2 rheometer (TA Instruments, New Castle, DE, USA) equipped with a concentric cylinder system for viscosity (cup diameter 20 mm or 30 mm depending on fluid tested) and a cone‐plate system (40 mm, 2.3° angle) for determination of normal force. The measurements were performed at 20°C.

### 2.6 Extensional Viscometry

The transient extensional viscosity of the thickened solutions was determined by the Hyperbolic Contraction Flow method (HCF) using an Instron 68SC‐5 (Instron Corp., Norwood, USA). The HCF method measures the force on a hyperbolic nozzle subjecting the sample to a constant extension rate and is previously thoroughly described and evaluated (Nyström et al. 2017; Stading and Bohlin 2000; Wikström and Bohlin 1999). The hyperbolic nozzle used for the measurement had an inlet radius of 10 mm and an outlet radius of 0.83 mm, which gives a total Hencky strain of typically 6.6–9.9 for the fluids investigated.

### 2.7 Density and Surface Tension

Density was measured using a Densito 30PX (Mettler Toledo, Schwarzenbach, Switzerland). Surface tension was determined by an OCA40 pendant drop instrument (DataPhysics Instruments, Filderstadt, Germany).

### 2.8 Statistics

Measured values are presented as mean values with error bars denoting the standard deviation. Three or more replicates were measured to calculate the mean values.

### 2.9 Theory

Dimensionless numbers are often used to estimate the influence of different physical mechanisms, here on the flow of fluids. The Ohnesorge number (Oh) is a dimensionless number that relates viscous forces to inertial and surface tension forces. A low Oh indicates droplet formation and fluid breakup for example, when a fluid exits a tube given by

(1) $$\mathrm{Oh}=\frac{\eta }{\sqrt{\mathrm{\rho \sigma D}}}$$

The Reynolds number (Re) is a dimensionless number that relates inertial forces to viscous forces and predicts laminar or turbulent flow as expressed for flow in a tube.

(2) Re=ρvDη

The Weissenberg number relates elastic forces to viscous forces in a viscoelastic fluid. In shear flow it can be expressed as follows:

(3) $$\mathrm{Wi}=\lambda \dot{\gamma }$$

In (Equations (1), (2), (3)), *η* denotes viscosity, *ρ* density, *σ* surface tension, *v* fluid velocity, *D* the tube diameter, *λ* the relaxation time and $\dot{\gamma }$ the shear rate.

## 3 Results and Discussion

### 3.1 Model Fluids

**FIGURE 2:**
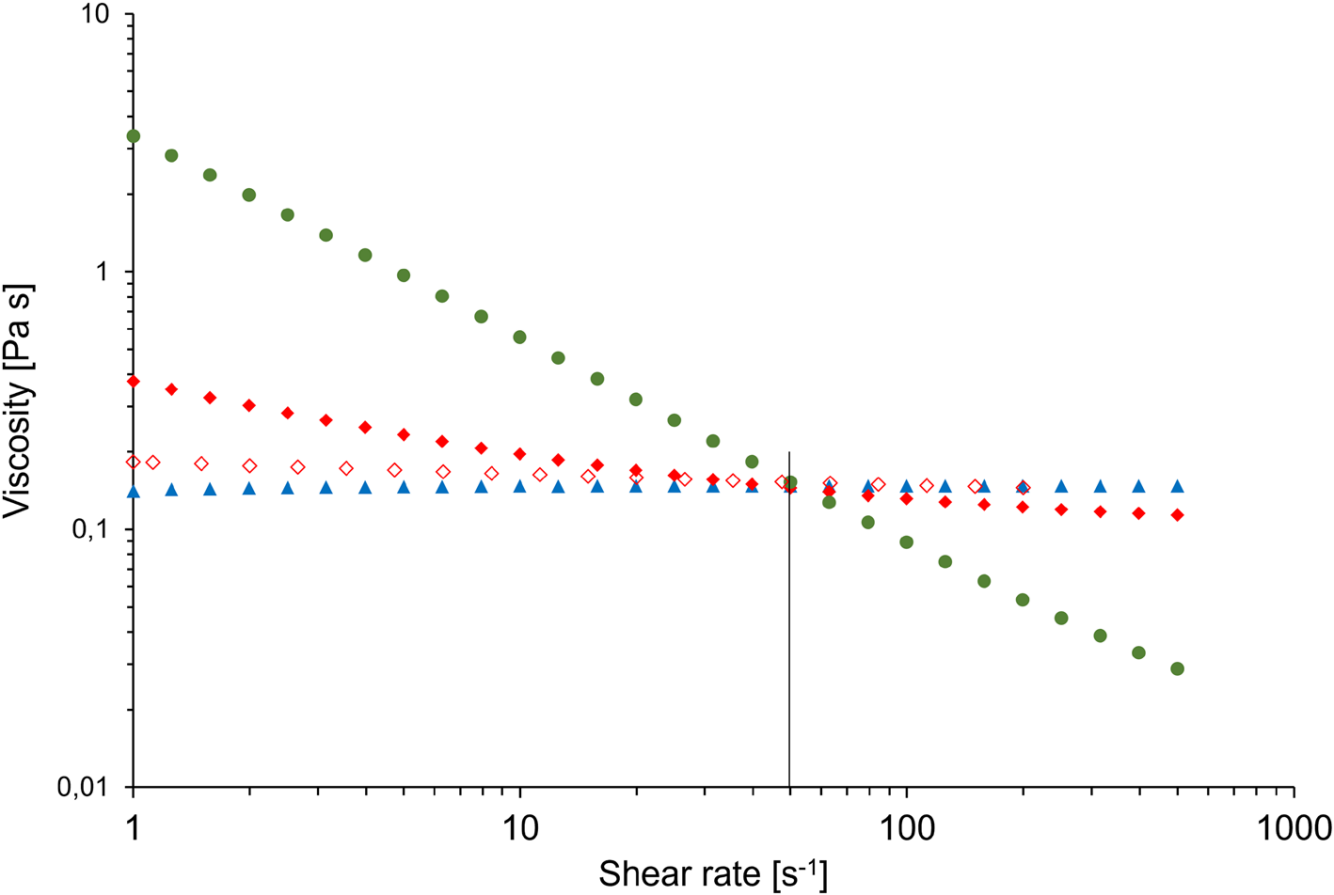
Viscosity as a function of shear rate for the model fluids studied, 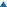 Newtonian, 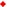 Boger_xanthan_, 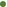 shear‐thinning. A Boger fluid with PAA instead of xanthan is shown for reference, 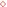. The solid line indicates 50 s^−1^. Error bars for the standard deviation were smaller than the symbols.

The flow curves for the three edible model fluids are shown in Figure 2. All were designed to have the same viscosity at 50 s^−1^ of 0.15 Pa s. The Boger fluid should ideally have the same constant shear viscosity as the Newtonian, but the limitation of using edible xanthan instead of for example, PAA makes it slightly shear‐thinning. The amount of added xanthan was optimized to be as low as possible, to maintain constant shear viscosity, while still giving a positive normal stress while sheared. The effect on viscosity of having a dilute lemonade instead of water as solvent was negligible, as the viscosity of the lemonade (viscosity = 1.02 ± 0.003 mPa s) was very close to that of water (1.0016 mPa s). A Boger fluid with PAA instead of xanthan was also prepared and measured to give a reference for a corresponding non‐edible Boger fluid. PAA is more elastic and gives a Boger fluid with a more constant shear viscosity as shown in Figure 2.

Similar model fluids have previously been prepared for other purposes (Koliandris et al. 2011) as well as for swallowing studies (Nyström et al. 2015). The concentrations and viscosity levels previously reported had the same magnitude, but variations between batches and suppliers make exact comparisons impossible.

**FIGURE 3:**
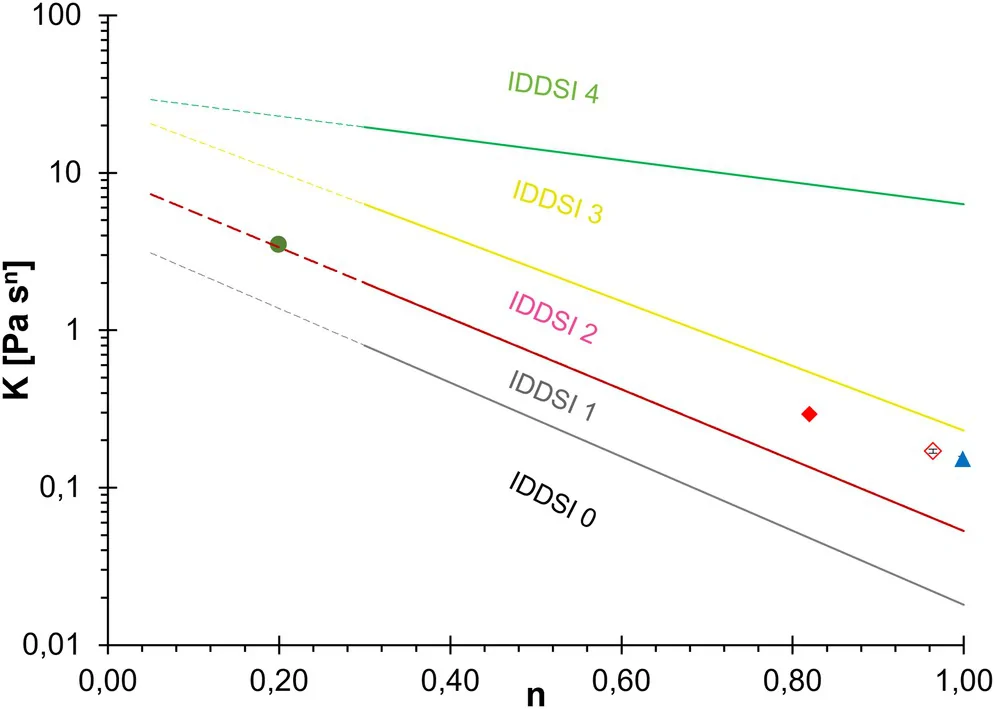
IDDSI levels adopted from Lecanu (Lecanu et al. 2024) with the three model fluids inserted: 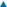 Newtonian, 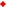 Boger_xanthan_, 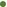 shear‐thinning. The dashed lines are extrapolations from the results by Lecanu. K and n are the Power Law parameters describing a shear‐thinning fluid (*n* < 1). A Boger fluid with PAA instead of xanthan is shown for reference, 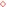. Error bars for the standard deviation were smaller than the symbols.

All model fluids had IDDSI 2 when measured using the IDDSI syringe test (Steele et al. 2024). The test uses a standardized syringe to measure remaining fluid after 10 s dripping. The rheology of this test has been translated to Power law parameters n and K obtained by viscometry (Lecanu et al. 2024). When plotting n and K in a diagram, the IDDSI levels appear as diagonal bands as shown in Figure 3. All three model fluids fall into the IDDSI = 2 band, even though the low viscosity of the shear‐thinning fluid pushes it toward the limit of IDDSI = 1.

The fluids were tested as is and no artificial saliva or saliva‐like buffer was added. In the INFOGEST protocol all samples are diluted with equal amounts of saliva buffer (Brodkorb et al. 2019). For boluses of solid food we have previously shown that this ratio is similar to what you find in real boluses (Stading 2021; Stading et al. 2023). For beverages it can be argued that there is less dilution by saliva as the ingestion is faster, but there are no quantitative results published from expectorated boluses.

As an estimation, a semi‐dilute xanthan solution has a viscosity, which is proportional to the concentration squared. A 50:50 dilution will thus reduce the viscosity to 0.5^2^ = 25% of the original viscosity. This would typically mean one step in IDDSI level as can be seen from Figure 3, for example, by comparing the IDDSI level for K and 0.25 K at *n* = 1. This is a qualitative discussion, but the point is that studying IDDSI level 2 fluids would correspond approximately to IDDSI 3 fluids diluted by saliva. Higher IDDSI levels are rarely relevant for beverages, and lower, that is, IDDSI 1, needs to be investigated to test if mainly their elastic properties are strong enough to contribute. Similarly, although the model fluids cover a significant part of the IDDSI 2 band, other thickeners like starch, and commercially available thickeners should be investigated as well.

**FIGURE 4:**
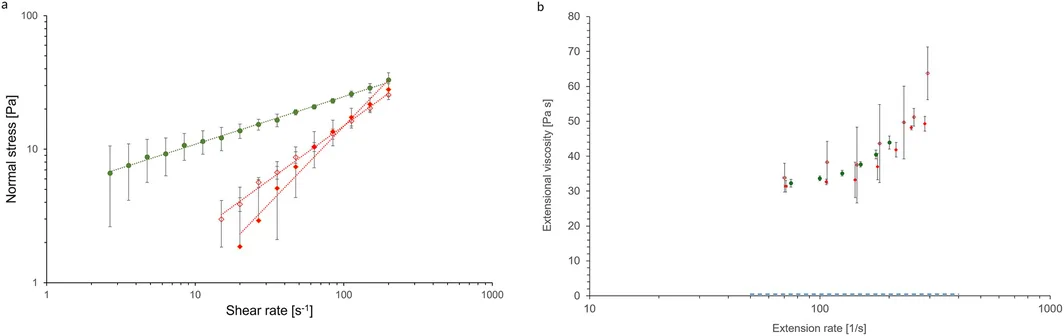
(a) Normal stress as a function of shear rate for the shear‐thinning and Boger fluids, and (b) extensional viscosity as a function of extension rate for the same fluids: 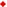 Boger_xanthan_, 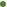 shear‐thinning. A Boger fluid with PAA instead of xanthan is shown for reference, 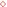. The dashed blue line for the Newtonian fluid indicates 3 × shear viscosity.

A positive normal stress during shear was used as an indication of elasticity in optimization of the Boger fluid. For low viscosity fluids, the normal stress is often overshadowed by inertia, and it is not an absolute measure of fluid elasticity. The normal stress of the Boger and shear‐thinning fluids is shown in Figure 4a. The shear‐thinning xanthan solution had lower shear rate dependence than Boger fluids, which can depend on several physical mechanisms. Xanthan forms a weak network, which easily can disentangle and thus build less elastic stress. The extended, non‐aligned chains at low shear rate mean slower relaxation, and the polymer contribution decreases with shear due to the shear‐thinning (Del Giudice et al. 2017; Zirnsak et al. 1999). In the Boger fluids, maltodextrin masks shear‐thinning.

Extensional viscosity is a more quantitative measure of fluid elasticity than the normal stress. The shear‐thinning and Boger fluids showed substantial extensional viscosity as shown in Figure 4b, especially compared to 3 × shear viscosity plotted for the Newtonian fluid. A Boger fluid containing PAA instead of xanthan is shown for reference, and it had the highest extensional viscosity due to the high elastic contribution of the PAA. A Trouton ratio could be calculated for the lowest extension rate and was in the range 5–90 for the fluids.

The method of Hyperbolic Contraction Flow determines the total measured stress on a hyperbolic nozzle through which the fluid is forced to extend. The stress is a sum of extensional and shear stresses, and the calculated shear stress is subtracted before calculating the extensional viscosity. For the Boger fluids, this means that only a small extensional component remains; thus, the higher standard deviation for the Boger fluids.

**FIGURE 5:**
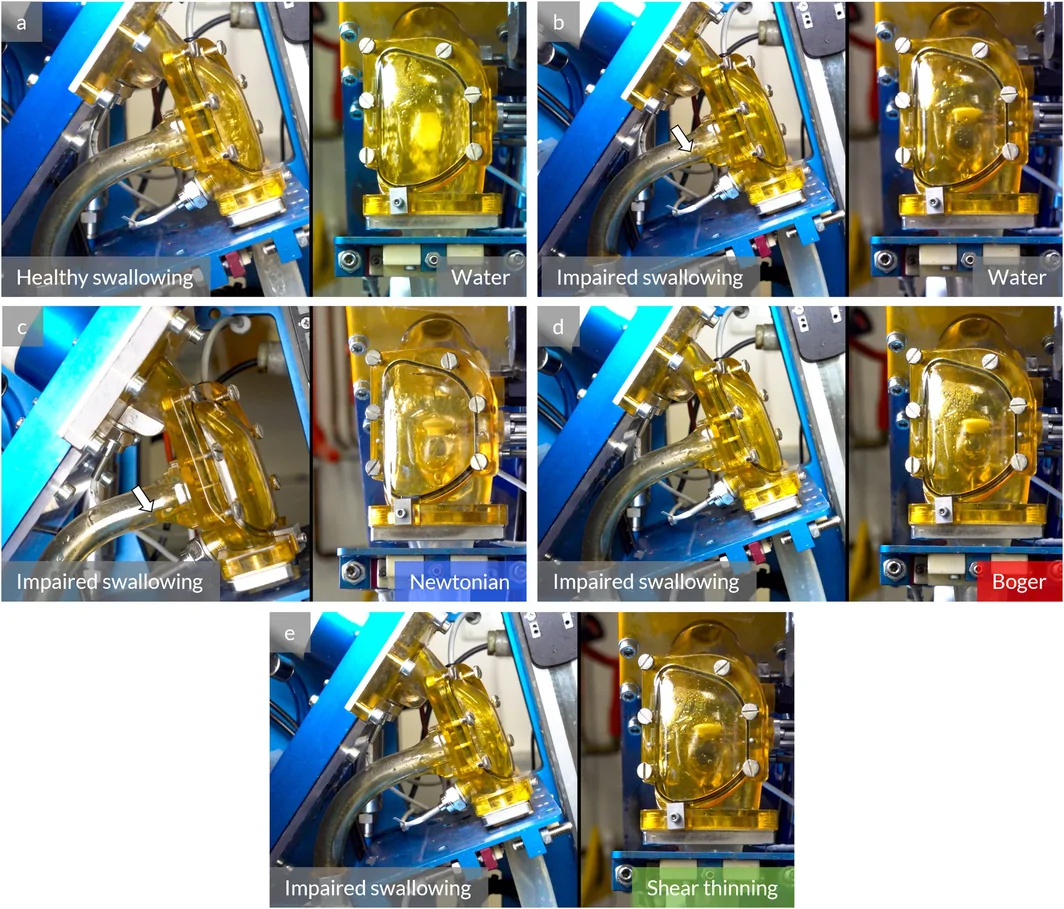
Snapshots of boluses passing the pharynx of the Gothenburg Throat model during bolus flow in side view (left) and front view (right). (a) Healthy swallowing of water, (b) impaired swallowing of water, (c) impaired swallowing of the Newtonian fluid, (d) impaired swallowing of the Boger fluid and (e) impaired swallowing of the shear‐thinning fluid. The arrows point to fluid entering the airways. The white moving part is the epiglottis. Videos of the swallowing events are found in the Supporting Information and in the Youtube play list https://youtube.com/playlist?list=PLqLiVcF3GKy2Cz3QHIjJB9NvDY2ZwAfid&si=42BmZfqwc51osNNj.

The flow during swallowing of the model fluids was evaluated using a model of the human throat, the “Gothenburg Throat” model (Stading et al. 2019), see Figure 1. The model can mimic healthy as well as impaired swallowing by setting the timing of opening and closing of the epiglottis, airways and the UES. The velocity measurements used in the model have previously been evaluated against video fluoroscopy (Qazi et al. 2020) and the same type of model fluids by a group of dysphagia patients (Nyström et al. 2015). The bolus volume was set to 30 mL which is relevant for drinking beverages although patients with severe dysphagia dysfunction would use smaller boluses (Colevas et al. 2022). The initial speed of the bolus set to 0.3 m/s is within the normal range of typically 0.1–0.5 m/s (Ekberg 2019; Qazi et al. 2019). For impaired swallowing the closing of the epiglottis was delayed 1 s and the airways and UES were open. Figure 5 shows the flow of the swallowed fluid bolus during passage through the pharynx. Videos in slow motion of the bolus flow is found in the Supporting Information and in the Youtube play list https://youtube.com/playlist?list=PLqLiVcF3GKy2Cz3QHIjJB9NvDY2ZwAfid&si=42BmZfqwc51osNNj.

The snapshots in Figure 5 show the bolus flow when most of the bolus has passed the pharynx, and the most interesting feature is flow into the airways (aspiration). Water is difficult to swallow for a person with dysphagia dysfunction as the flow is turbulent and the bolus splits up in droplets. When comparing Figure 5a,b, there is water in the airways for the impaired swallowing in Figure 5b, but not in 5a. If the fluid is thickened from 1 mPa s of water to 0.15 Pa s with maltodextrin, there is still flow into the airways as shown in Figure 5c, which means that although higher viscosity, the Newtonian fluid still causes aspiration. When thickening the fluid with maltodextrin and xanthan (Boger fluid) there is no flow into the airways as shown in Figure 5d. The Newtonian and Boger fluids have the same shear viscosity, but the Boger fluid is substantially more elastic due to the addition of xanthan. Similarly, Figure 5d shows that the shear‐thinning fluid also prevents flow into the airways. The conclusion so far is that both elasticity and shear‐thinning are helpful to prevent aspiration.

**FIGURE 6:**
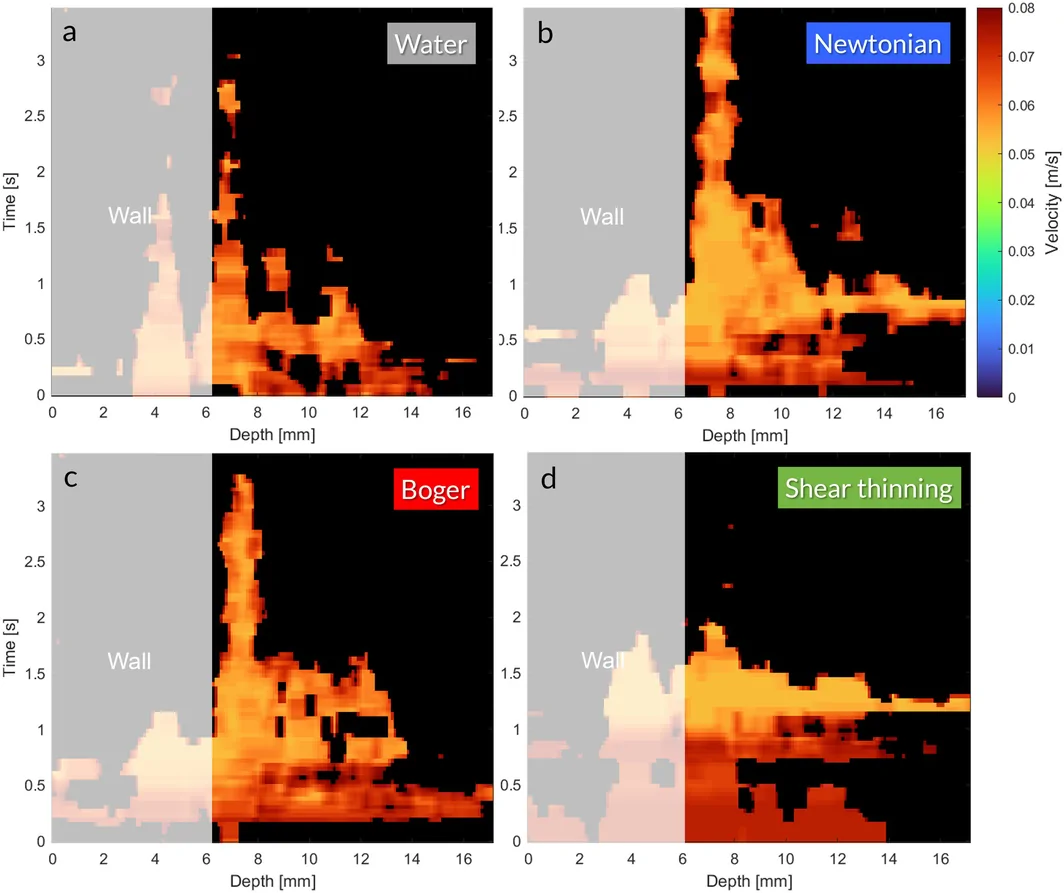
Velocities of boluses shown by the color scale, of the model fluids along a beamline through the center of the pharynx (see Figure 1), for increasing time of flow. (a) water, (b) Newtonian fluid, (c) Boger fluid, and (d) Shear thinning fluid.

Figure 6 shows the bolus flow of water and the model fluids in more detail. The colors denote the velocity of the bolus at a specific point along a line from the transducer into the pharynx, that is, depth is plotted on the horizontal x‐axis. The transducer was placed just above the entrance to the trachea. Time is on the vertical y‐axis, which means that the front of the bolus is close to the x‐axis and the delayed flow is higher on the y‐axis. The velocity‐time curves are averages of at least three measurements.

The fastest flow occurs early in time and close to the wall, which also is obvious from the videos of the bolus flow. Figure 6a clearly shows that the water bolus breaks up in parts indicated by the separated peaks, contrary to the other fluids where the bolus is more cohesive.

When comparing the bolus flow of the Newtonian fluid (Figure 6b) with that of water (Figure 6a), the Newtonian fluid is more cohesive and does not break up in droplets. It is still not sufficiently cohesive to prevent flow into the airways. Flow continues close to the wall over the whole measured period, which is in line with the Newtonian character of the fluid.

The Boger fluid (Figure 6c) is spatially more cohesive than the Newtonian bolus although flow continues close to the wall. The small improvement in cohesiveness is, however, sufficient to avoid flow into the airways. The shear‐thinning bolus is even more cohesive and has less flow over time along the wall, which may be explained by the shear‐thinning behavior, which results in a higher apparent viscosity at low shear rates.

## 4 Bolus Cohesivity

Dimensionless numbers are commonly used in fluid mechanics to quantify complex flow behavior and to compare fluids with different properties. In this context they can be used to quantify the cohesivity of the bolus. Although a loose term, we here relate cohesivity to the breakup of the bolus into smaller drops, which is a structural definition that has functional implications such as increased risk of aspiration. Please note that bolus cohesivity has nothing to do with “cohesivity” used in other areas to describe stickiness such as for adhesives and in texture profile analysis. The main goal of thickening is to slow the flow of the bolus to give the impaired physiology time to react. The flow through the pharynx is a complex flow and in addition to slowing the flow, thickening also affects bolus cohesivity. The forces acting on the bolus are viscous, elastic, and inertial in nature, and surface tension plays an important role in droplet formation. The relevant parameters of the fluid passing through the pharynx are thus viscosity, velocity, the relaxation time, density, and the surface tension. These were determined for the model fluids, and the relaxation times were taken from literature, and are presented in Table 1.

**TABLE 1:** Physical data of the model fluids. A pharynx width of 2.5 cm and velocity of 0.1 m/s through the pharynx were used for calculating the Reynolds and Ohnesorge numbers.

	Fun Light	Newtonian	Boger	Shear‐thinning
Density [kg/m^3^]	1000	1240	1220	1000
Viscosity at 50 s^−1^ [Pa s]	0,001	0,15	0,15	0,15
Surface tension [N/m]	0,058	0,052	0,051	0,057
Relaxation time [s]	1E‐11	0,001	0,5	1
Reynolds number	2500	21	20	17
Ohnesorge number	0,00093	0,13	0,13	0,14
Weissenberg number	5E‐10	0,05	25	50
Ohnesorge × Weissenberg/Reynolds	2E‐16	3E‐04	0,17	0,42

The longest relaxation time in the shear‐thinning fluid (0.45% xanthan) could be estimated from previously published results to 0.3–3 s (Berta et al. 2018; Choppe et al. 2010). The longest relaxation time of the Boger fluid could be estimated from a similar system, xanthan in syrup (Zirnsak et al. 1999) where it was found to be 0.3–3 s. Compensating for the relatively higher viscosity of the syrup system compared to the 50% maltodextrin fluid, scaling arguments would push the relaxation times more to 0.1–1 s (Sousa et al. 2017). The relaxation time of water (here Fun Light) at room conditions is very low, in the order of picoseconds. The exact value can be discussed, but for this context it is enough to conclude that it is much smaller than that for the model fluids. The addition of the lemonade caused the surface tension to drop from that of water at 20°C of 0.072 N/m to 0.051–0.058 N/m. The maltodextrin and xanthan may also have a small effect.

The Reynold's number predicts whether a flow is laminar or turbulent or transitional, that is, a mix between the two. The regimes can be expressed in terms of the Reynold's number as laminar for Re < 2300, transitional for 2300 < Re < 4000 and turbulent for Re > 4000. All model fluids displayed laminar flow and Fun Light transitional which correlates well with the observed flow shown in Figure 5.

The Ohnesorge number is mainly used to analyze free‐surface flows to predict droplet breakup as when the fluid passes the epiglottis where the channel is only partly filled. A low Ohnesorge number indicates droplet breakup whereas a high number indicates high viscosity and stable flow resisting breakup. The model fluids have similar Ohnesorge numbers due to having the same viscosity at 50 s^−1^ and similar surface tension and densities, as opposed to Fun Light where droplet breakup is evident (c.f. Figure 5). As the model fluids do not all have the same constant viscosity, the Ohnesorge number does not represent the full picture.

The Weissenberg number relates elastic to viscous forces and shows a difference between Newtonian as compared to the Boger and the Shear‐thinning fluids. For Fun Light it is very low primarily due to the lack of elasticity. The Weissenberg number clearly shows the effect of adding xanthan to the maltodextrin, that is, going from Newtonian to Boger behavior, which likely is why the Boger fluid does not enter the airways as opposed to the Newtonian fluid (Figure 5).

There is no clear indication on bolus cohesivity from a single dimensionless number, but by multiplying the two numbers promoting bolus cohesivity, Ohnesorge × Weissenberg and dividing by the Reynolds number that impairs cohesivity, a “bolus cohesivity number” could be formed:

(4) Bolus cohesivity number=OhxWiRe

As viscous and inertial forces are part of several of the dimensionless numbers, the bolus cohesivity number is linearly dependent on elastic and viscous forces and inversely proportional to surface forces and inertial forces squared. Table 1 shows a clear difference in the bolus cohesivity number, especially for the Boger and Shear‐thinning fluids as compared to the Newtonian fluid and to Fun Light, and it correlates well with the observed differences in fluid entering the airways. A high bolus cohesivity number means safe swallowing in that aspiration is avoided. The physical interpretation of the bolus cohesivity number is that it describes how well a bolus keeps together when it is subjected to elastic, viscous, inertial, and surface forces.

## 5 Conclusions

The primary objective for thickening of beverages for dysphagia management is to slow down the flow to give the impaired physiology time to react and perform the necessary functions of the pharynx, to close the opening to the airways by the epiglottis, to keep the breath by closing the vocal cords, and to open the UES. A further objective is to alleviate or prevent aspiration. Our results show thickening can be achieved in several ways, and that the mechanism of thickening matters for how effective the fluid is to prevent aspiration.

From a nutritional perspective, it may be tempting to thicken a fluid with maltodextrin as it is then possible to add much energy in the form of carbohydrates yet keeping viscosity at a reasonable level. However, the results show that this results in a Newtonian fluid, and Newtonian thickening is less effective than shear‐thinning or elastic mechanisms to avoid aspiration. The results further show that adding elasticity (Boger fluid) induces cohesiveness to the bolus, thus preventing the aspiration a Newtonian fluid with the same shear viscosity shows. Cohesiveness is further improved in the shear‐thinning fluid, which in addition to the shear‐thinning character, also is elastic.

By using a combination of dimensionless numbers for a Bolus cohesivity number the cohesivity can be quantified, with a high number indicating cohesivity. This is a preliminary hypothesis which needs to be evaluated on larger data sets for more fluids, but it correlates well with observed cohesivity.

The limited experimental data set should be extended, but the results on the important mechanisms are still clear. The set of model fluids should also be evaluated sensorily regarding swallowing using panels of older adults who preferably have or can detect dysphagia symptoms. From a dysphagia management perspective, many of the commercially available thickeners are already based on xanthan which is positive, but the results open up opportunities for further improvement.

## Author Contributions

**Mats Stading:** conceptualization, investigation, funding acquisition, writing – original draft, methodology, validation, visualization, formal analysis, project administration, data curation. **Johanna Eckardt:** methodology, writing – review and editing, data curation, investigation.

## Funding

This work was supported by Svenska Forskningsrådet Formas, AC‐2024/0018.

## Ethics Statement

The authors have nothing to report.

## Conflicts of Interest

The authors declare no conflicts of interest.

## Supporting information


**Figure 5a** Video: Swallowing water, healthy, 10× slowmotion.

**Figure 5b** Video: Swallowing water, dysphagia,10× slowmotion.

**Figure 5c** Video: Swallowing Newtonian fluid, dysphagia,10× slowmotion.

**Figure 5d** Video: Swallowing Boger fluid, dysphagia,10× slowmotion.

**Figure 5e** Video: Swallowing shear thinning fluid, dysphagia,10× slowmotion.

## Data Availability

Research data are not shared.
